# Correlation Analyses of Clinical Manifestations and Variant Effects in *KCNB1*-Related Neurodevelopmental Disorder

**DOI:** 10.3389/fped.2021.755344

**Published:** 2022-01-05

**Authors:** Juan Xiong, Zhonghua Liu, Shimeng Chen, Miriam Kessi, Baiyu Chen, Haolin Duan, Xiaolu Deng, Lifen Yang, Jing Peng, Fei Yin

**Affiliations:** ^1^Department of Pediatrics, Xiangya Hospital, Central South University, Changsha, China; ^2^Hunan Intellectual and Developmental Disabilities Research Center, Changsha, China; ^3^The National and Local Joint Engineering Laboratory of Animal Peptide Drug Development, College of Life Sciences, Hunan Normal University, Changsha, China

**Keywords:** *KCNB1*, neurodevelopmental disorder, loss of function, dominant-negative effect, haploinsufficiency

## Abstract

**Objective:** *Vitro* functional analyses of *KCNB1* variants have been done to disclose possible pathogenic mechanisms in *KCNB1*-related neurodevelopmental disorder. “Complete or partial loss of function (LoF),” “dominant-negative (DN) effect” are applied to describe *KCNB1* variant's molecular phenotypes. The study here aimed to investigate clinical presentations and variant effects associations in the disorder.

**Methods:** We reported 10 Chinese pediatric patients with *KCNB1*-related neurodevelopmental disorder here. Functional experiments on newly reported variants, including electrophysiology and protein expression, were performed *in vitro*. Phenotypic, functional, and genetic data in the cohort and published literature were collected. According to their variants' molecular phenotypes, patients were grouped into complete or partial LoF, and DN effect or non-dominant-negative (non-DN) effect to compare their clinical features.

**Results:** Nine causative *KCNB1* variants in 10 patients were identified in the cohort, including eight novel and one reported. Epilepsy (9/10), global developmental delay (10/10), and behavior issues (7/10) were common clinical features in our patients. Functional analyses of 8 novel variants indicated three partial and five complete LoF variants, five DN and three non-DN effect variants. Patient 1 in our series with truncated variants, whose functional results supported haploinsufficiency, had the best prognosis. Cases in complete LoF group had earlier seizure onset age (64.3 vs. 16.7%, *p* = 0.01) and worse seizure outcomes (18.8 vs. 66.7%, *p* = 0.03), and patients in DN effect subgroup had multiple seizure types compared to those in non-DN effect subgroup (65.5 vs. 30.8%, *p* = 0.039).

**Conclusion:** Patients with *KCNB1* variants in the Asian cohort have similar clinical manifestations to those of other races. Truncated *KCNB1* variants exhibiting with haploinsufficiency molecular phenotype are linked to milder phenotypes. Individuals with complete LoF and DN effect *KCNB1* variants have more severe seizure attacks than the other two subgroups.

## Introduction

*KCNB1* encodes α-subunit of the Kv2.1 channel, which consists of six transmembrane segments (S1–S6), with a voltage-sensing domain (VSD, S1–S4) and ion-conducting pore formed by domain S5 and S6 (1). *In vivo*, homotetrameric Kv2.1 channels (assembled only by α-subunits) contribute a large portion of delayed rectifier potassium currents in various neuron subtypes, including pyramidal neurons of the hippocampus and cortex (2, 3), while heterotetrameric Kv2.1 channels (co-assembled with α and β-subunits) are also involved in physiological processes such as regulating excitability in mouse dorsal root ganglia neurons (4–6). Since Torkamani et al. (7) firstly correlated epileptic encephalopathy with *KCNB1* variants in 2014, about 80 patients with *KCNB1*-related neurodevelopmental disorders have been reported due to extensive clinical genetic testing (8, 9). Now, the gene is one of the most mutated potassium genes in children with neurodevelopmental disorders. Most patients with *KCNB1*-related neurodevelopmental disorders have variable epilepsy, intellectual disabilities, psychiatric and behavior problems (9, 10).

Most functional studies *in vitro* focused on the potassium conductance properties of KCNB1 protein. Researchers have successfully applied high-through automated electrophysiology to analyze the pathogenicity of *KCNB1* variants (11). Nevertheless, the differences between the automated and manual methods in the channel have never been studied. Unlike *in vivo*, assembling Kv2.1 channels with solely *KCNB1* wild-type or variant subunits is considered homomeric configuration, while co-assembling with wild-type and variant is considered heteromeric configuration *in vitro* (11, 12). Now, it has been proven that causative *KCNB1* variants result in loss of Kv2.1 channel function, which is classified into “partial loss of function (LoF)” and “complete LoF” according to the amplitude of Kv2.1 currents in homotetrameric expression models (7, 11–13). Like other genetic variants of ionic channels (14), the dominant-negative (DN) phenomenon that variants could interfere with the co-expressed wild type, has also been confirmed as the pathogenesis of the disorder (15). Conversely, those variants that do not have sufficient evidence to support the DN phenomenon could be subgrouped as the non-dominant-negative (non-DN) effect. However, what genetic mechanisms underlying them are unclear. Notwithstanding, whether patients could benefit from the above functional classification remains unknown.

Here, we described detailed clinical and genetic information of 10 patients with *KCNB1*-related neurodevelopmental disorders from the Chinese population [1 had been described briefly before (16)]. Functional experiments, including manual electrophysiology and western blotting of eight novel *KCNB1* variants, were done *in vitro* to disclose their functional alterations. Combined with previously published phenotypic, functional, and genetic data of the disorder, we linked variant effects to clinical presentations, which was innovative attempts to study the disease.

## Materials and Methods

### Individuals

Children with *KCNB1*-related neurodevelopmental disorders were enrolled in the study at Xiangya Hospital, Central South University from May 2013 to January 2021. Detailed clinical data of all patients were evaluated by two experienced pediatric neurological doctors, including perinatal history, family history, developmental history, seizure types, neuropsychiatric symptoms, genetic results, video electroencephalography (EEG), neuroimaging, developmental scales, and therapeutic schedules. Follow-up work was undertaken by outpatient or phone call.

### Variant Analysis

Next-generation sequencing of epilepsy panel (17) for 2 cases and whole-exome sequencing for other 8 patients was adopted in the cohort (Table 1). Candidate variants were validated by Sanger sequencing. The data were analyzed as described in previous studies (17, 18). The pathogenicity of these variants was interpreted according to the American College of Medical Genetics (ACMG) guidelines (19).

**Table 1:** Clinical characteristics and genetic information of patients with *KCNB1* related-neurodevelopmental disorder reported in our cohort.

**Patient No**.	**Variant**	**Testing Methods**	**Current Age /Sex/Age at Seizure Onset**	**Epilepsy/Age at** **Seizure Offset**	**Therapeutic Schedules**	**DD**	**Behavior problems**	**MRI Findings**	**EEG findings**	**Neurologic Examination Findings**	**Other**
1	chr20:49374989 c.571delC p.A192Pfs*1 exon 2	WES	8.2 y/F/5 m	IS, S/10 m	LEV/ACTH	Normal before seizure, mild-moderate at infancy, now in regular school	N	N	Hypsarrhythmia, turned into normal at 6 y	N	N
2	chr20:49374931 c.629C > T p.T210M exon 2	WES	5.3 y/M/7 m	Febrile seizures, 3 times in all	-	Observed at 6 m, moderate- severe, walk at 17 m, single words	N	Slightly larger left lateral ventricle	Rolandic discharges	N	N
3	chr20:49374931 c.629C > T p.T210 M exon 2	WES	14.9 y/F/12 m	Febrile seizures between 2 and 6 y, clusters eyelid MS from 9 y, occasional GTCS at 13 y/ ongoing, almost every day	NZP/VPA	Noticed at 18 m, moderate- severe, simple communication, cannot read, able to run	Hyperactivity, stereotypies, irritability	N	Multifocal, generalized discharges, photosensitive	Ataxic gait	N
4	chr20:47991283 c.C814T p.P272S exon 2	Epilepsy Panel	12.1 y/M/20 m	GTCS, atypical Ab, FS, related to fever/ongoing, 5–6/year	LEV/OXC/ NZP/VPA/ LTG/CLB/KD	Observed at 12 m, severe at childhood, walked dependently at 2 y, simply express needs, write his own name	Irritability, aggression	N	Slow background, multifocal discharges, CSWS	Ataxic gait, hypotonia	Inguinal hernia, sleep troubles
5	chr20:47991157 c.T940C p.S314P exon 2	WES	4.5 y/M/6 m	GTCS, several episodes during half years, triggered by fever/12 m	LEV	Noticed at 6 m, moderate-severe, non-verbal	Irritability, aggression	N	diffuse and multifocal discharges	Ataxic gait	Dysphagia
6	chr20:47991143 c.G954T p.Q318H exon 2	WES	7.0 y/M/30 m	FS, GTCS, several episodes in 1^st^ year, controlled at 44 m; recurred at 64 m/65 m	LEV/NZP	Noticed at 6 m, severe, able to walk, run, several words	Irritability, hyperactivity	N	Slow background, multifocal discharges, CSWS	Ataxic gait	Hyperchromic skin spot on abdomen
7	chr20:47991107 c.G990T p.E330D exon 2	WES	3.1 y/M/8 m	GTCS, several episodes in the first 3 months, related to fever /11 m	LEV	Observed at 6 m, moderate-severe, cannot walk, single words	N	N	Multifocal discharges	Hypotonia	N
8	chr20:47990967 c.C1130T p.T377I exon 2	WES	3.7y/M/6m	IS, S MS, AS, GTCS clusters, occurs every day/ongoing, “honeymoon period” with OXC	ACTH/TPM /LEV/VBG /KD/VPA /OXC	Observed at 3m, profound, cannot sit, no words	Stereotypic hand movements	Smaller right hippocampus	Slow background, multifocal, generalized discharges, hypsarrhythmia	Hypotonia	Laryngeal obstruction; recurrent infections
9	chr20:47990961 c.G1136T p.G379V exon 2	WES	9.3 y/M/13 m	IS turned into LGS, S, AS, MS, GTCS, /ongoing, “honeymoon period” with steroid and NZP	LEV/MPS/ TPM/ KD/OXC/ MPS/NZP	Noticed at 6 m, profound, non-verbal	Autistic like traits	N	Slow background, generalized discharges, CSWS	Hypotonia, ataxic gait	N
10	chr20:47990875 c.G1222A p.P408S exon 2	Epilepsy Panel	7.0 y/F/11 m	GTCS, FS, atypical Ab, related to fever or infection/ongoing, 1–2/month	VPA/ LEV/OXC	Observed at 3 m, severe, only can walk, non-verbal	Stereotypic hand movements, irritation	N	Slow background, multifocal, generalized discharges	Ataxic gait	Recurrent infections

*Ab, absence seizures; ACTH, Adrenocorticotropic Hormone; AS, atonic seizure; CLB, clobazam; CSWS, continuous spikes and waves during slow-wave sleep; DD, developmental delay; EEG, electroencephalogram; F, female; FS, focal seizures; GTCS, generalized tonic–clonic seizures; IS, infantile spasm; KD, Ketogenic diet; LEV, levetiracetam; LGS, Lennox-Gastaut syndrome; LTG, lamotrigine; M, male; m, month; MPS, methylprednisolone; MRI, magnetic resonance imaging; MS, myoclonic; N, no; NZP, Nitrazepam; OXC, oxcarbazepine; S, spasm; TPM, topiramate; VBG, vigabatrin. VPA, valproate; WES, whole-exome sequencing*.

### Functional Analyses

#### Whole-Cell Electrophysiology

According to the manufacturer's instructions, the variant was introduced into the human KCNB1 cDNA (NM_004975) cloned into the pcDNA3.1 vector by site-directed mutagenesis (TOYOBO, Osaka, Japan). A similar transfection protocol was adopted to represent homomeric and heteromeric configurations (11). The total DNA amount was 2ug. Currents from CHO-K1 cells were recorded using an EPC-10 USB patch-clamp amplifier operated by PatchMaster software (HEKA Elektronik, Lambrecht, Germany) room temperature (20–22°C) 24–48 h after transfection. Bath solution contained (in mmol/L) 138 NaCl, 5.4 KCl, 2 CaCl_2_, 1 MgCl_2_, 10 glucose and 10 HEPES (pH 7.4 with NaOH); pipette solution contained (in mmol/L): 140 KCl, 2 MgCl_2_, 10 EGTA, 10 HEPES, 5 Mg-ATP (pH 7.4 with KOH).

The holding potential was−80 mV. A family of square voltage steps consisting of 500 ms depolarizing pulses from−80 mV to +120 mV (10 mV steps) followed by a 100-ms step to−80 mV (tail-currents) was applied. The peak current was measured from−80 mV to +60 mV, normalized for cell capacitance, then plotted against voltage to generate current density–voltage relationships. Voltage-dependence of activation was built-up by plotting tail currents normalized to the largest tail current amplitude against the corresponding test potential and fit with the Boltzmann equation. The cells were depolarized from−80 to +50 mV for 2.0 s, followed by an isopotential pulse at +60 mV of 500 ms duration to generate voltage-dependence of inactivation curves. The current values recorded at the beginning of the +60 mV pulse were measured, normalized, and fit to a Boltzmann distribution.

### Protein Extraction and Western Blotting

Minute^TM^ Plasma Membrane Protein Isolation and Cell Fractionation Kit (Invent, Plymouth, USA) were adopted to separate the total and plasma membrane KCNB1 proteins. We collected the total protein fractions before cellular component separation and isolated plasma membrane proteins according to the manufacturer's protocol. The proteins of CHO-K1 co-expressing variants and wild-type were extracted using ice-cold RIPA buffer with a ratio of 100:1 protease inhibitor cocktails (Beyotime, Shanghai, China). The total amount of plasmid DNA here maintained 4 ug.

20 ug of samples of proteins were separated by 10% sodium dodecyl sulfate– polyacrylamide (SDS-PAGE), transferred to PVDF membranes, blocked in protein free rapid blocking buffer (Epizyme, Shanghai, China), and incubated with mouse anti-Kv2.1 (1:1000; Abcam, ab192761), mouse anti-transferrin receptor (1:500; Invitrogen, #13-6800), rabbit anti-GAPDH antibody (1:2000; Servicebio, GB11002) overnight at 4 °C, then incubated with peroxidase-conjugated goat anti-rabbit IgG (1:10,000 Jackson ImmunoResearch) and goat anti-mouse IgG (1:10,000, Jackson ImmunoResearch) (1h at room temperature). Protein quantitation was completed using ImageJ software (NIH, Bethesda, USA).

### Literature Review

PubMed literature search was conducted by combining “*KCNB1*” or “Kv2.1 channel” and “variants” or “mutations” until 16 July 2021. Articles with functional studies of *KCNB1* variants were reviewed carefully. A similar method in previous research was used to classify partial LoF and complete LoF variant (11, 12). *In vitro* heteromeric expression models, variants that exhibited adverse effects on current amplitudes, or channel kinetics, or protein expression of Kv2.1 wild-type subunit were classified as DN effect variants, otherwise grouped as non-DN variants. Variants with controversial conclusions or unclear classifications were excluded. We analyzed published patients carrying enrolled variants with sufficient clinical information and grouped them to compare their clinical features.

### Statistical Analysis

The analysis and display of patch-clamp and western blotting data was performed using GraphPad Prism 8. A one-sided Fisher's exact test with *p* < 0.05 determining statistical significance in analyzing clinical features including seizure and intellectual disabilities in different subgroups (“complete LoF” subgroup vs. “partial LoF” subgroup, “DN effect” subgroup vs. “non-DN effect” subgroup).

## Results

### Clinical Description and Prognoses of 10 Newly Reported Patients

Ten unrelated neurodevelopmental disorder patients with *KCNB1* variants, including three females, and seven males, were recruited in the study (Table 1). Except for patient 2 with febrile seizures, the rest were diagnosed with epilepsy. The seizure onset age ranged from 5 to 30 months (median age, 11.5 months), and 6/9 of the patients' epileptic events were related to fever. Generalized tonic-clonic seizures (8), spasms (3), myoclonic seizures (3), focal seizures (3), atonic seizures (2), and atypical absence (2) were observed in our patients. Six cases had two or more seizure types. Three cases were diagnosed as infantile spasms, and one turned into Lennox-Gastaut syndrome. The age at the final follow-up ranged from 3.1 to 14.7 years old. Out of those 9 cases, 4 achieved seizure freedom from 10 to 65 months (median age, 11.5 months). The remaining 5 cases had drug-resistant epilepsy (3 cases with daily seizures and 2 with monthly seizures). Five of the 9 cases were treated with levetiracetam as the initial antiepileptic drug, which was effective for 4 cases. Three patients tried nitrazepam as add-on therapy and obtained seizure reduction. One of the two cases with infantile spasms was responsive to ACTH. Two of the three cases that tried ketogenic diet had significant seizure reductions and cognitive improvements, with more verbal expression in case 4, and more flexible eye contact in case 8.

All patients presented with global developmental delay; Eight displayed before seizures onset while patient one experienced developmental regression after epilepsy. The first noticeable symptom was stunted motor skill development. At the last follow-up, only patient one showed average intelligence level. Eight of the nine cases could walk dependently, but with ataxic gait, patient 7 could walk with support at the age of 2.4 years, and patient 8 could not raise his head at the age of 3.0 years. Patient 1 had a mild speech impediment in her infancy. She could not say “Mam/Dad” until 18 month-old age but now communicate with others normally. Of the rest nine patients, four had no words, two could speak single word, one could say several words, and two could communicate simply with short sentences. Of the 10 cases, seven individuals had behavioral problems, including autistic features in Four, hyperactivity in three, and aggression in three.

Video EEG of all patients showed abnormal profiles. Slow background activity was noticed in 5 out of 10 patients. Continuous spikes and waves during slow-wave sleep (CSWS) was detected in three patients, and hypsarrhythmia was found in two. Diffuse or generalized spike waves and polyspike waves were observed in five patients. Multifocal epileptiform discharges were recorded in seven patients.

### Nine *KCNB1* Variants Were Identified in Our Cohort

Nine *KCNB1* variants in our cohort were found, including 8 missense variants (p.T210M, p.P272S, p.S314P, p.Q318H, p.E330D, p.T377I, p.G379V, and p.P408S), and 1 in-frame deletion (p.A192Pfs^*^1) (Table 1). Eight of them were novel. It is worth noting that variant p.E330D detected here is caused by the 990^th^ base substituted by T, instead of the previously reported C (11.20). p.T210M has been identified in four cases except our two patients, which was a hotspot variant^8, 10, 11, 21^. According to ACMG guidelines, three variants (p.A192Pfs^*^1, p.T210M and p.E330D) were classified as pathogenic, and five (p.P272S, p.S314P, p.Q318H, p.G379V, and p.P408S) were likely pathogenic variants and one (p.T377I) was uncertain significance variant. These variants were distributed sporadically in KCNB1 protein, two of which were in S4–S5 linker (p.Q318H and p.E330D), two in S5-S6 linker (p.T377I and p.G379V), and one respectively in domain S1 (p.A192Pfs^*^1), S1–S2 linker (p.T210M), S3 (p.P272S) and S6 (p.P408S).

### Functional Analyses of the Detected *KCNB1* Variants in the Cohort

Except for p.T201M, whose functional alteration had been disclosed (11), *vitro* experiments on eight identified novel variants were undertaken in the study. Three partial LoF variants were identified, while one of them, variant p.E330D, was classified as complete LoF in Kang's study; the DN effects of 5 variants were confirmed, including variant p.E330D, whose result here was similar to that in Kang's study (11).

### Current Density, the Voltage Dependence of Channel Activation and Inactivation

**Figure 1:**
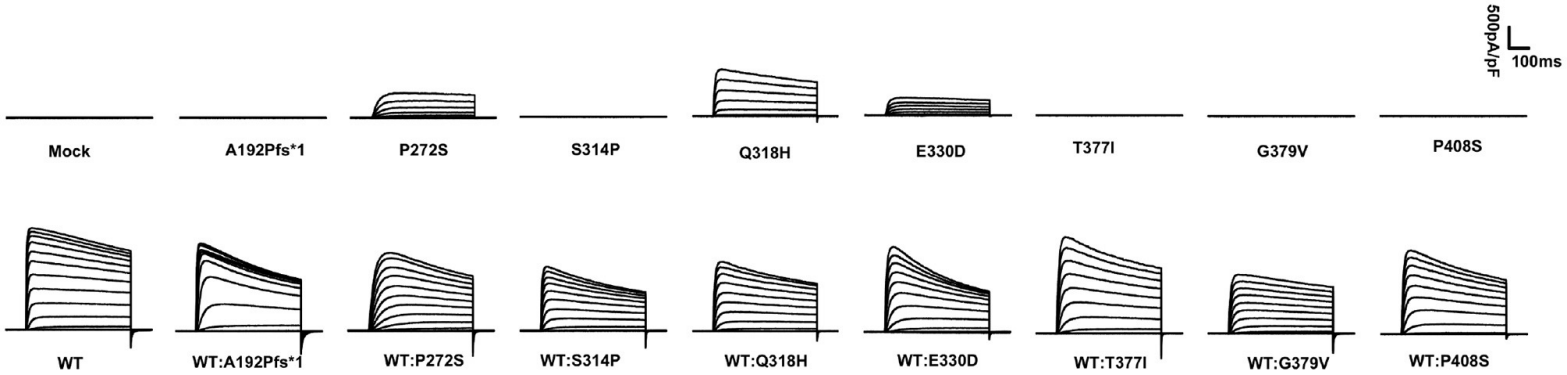
Averaged whole-cell current density traces of CHO-K1 transiently expressing MOCK or *KCNB1* subunits.

**Figure 2:**
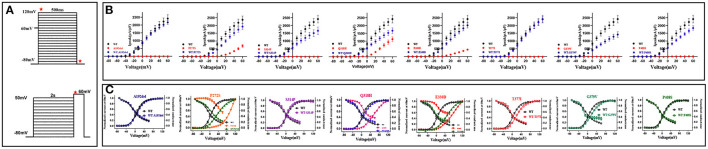
Biophysical properties of *KCNB1* variants detected in the study. **(A)** The voltage protocols used for electrophysiological studies are depicted. In the above image, Ipeak densities of *KCNB1* variants were recorded at a voltage from−80 to +60 mV, activation curves were generated at a voltage from−80 to +120 mV. Inactivation curves were recorded as the below figure. Red stars indicate time points of the current measurement. **(B)** Comparison of Ipeak density of *KCNB1* variants in homomeric and heteromeric configurations. **(C)** Voltage-dependence of activation/inactivation curves of *KCNB1* variants in homomeric and (or) heteromeric models.

The display of representative whole-cell current density traces of CHO-K1 cells that are transiently expressing mock, wild-type, or variants in Figure 1. The voltage protocols used here for electrophysiology studies are depicted in Figure 2A. The current density comparisons of homomeric and heteromeric channels of all variants are shown in Figure 2B. Variants p.P272S, p.Q318H, and p.E330D were identified as partial LoF variants, while the other five were complete LoF here, for no detectable outward potassium current were recorded. When co-expression with the wild-type subunits, the peak current density of variants p.A192Pfs^*^1, p.P272S, p.E330D, p. T377I, and p.P408S, significantly increased to >75% of which in cells of wild-type. In contrast, other variants amounted to 50%-75% relative to wild-type, and variant p.G379V seemed to have a dominant-negative effect on the magnitude of wild-type current. The volume and number of CHO-K1 cells included for analyzing the current density of each group are listed in Supplementary Table 1.

Voltage-dependence of channel activation and inactivation of these variants were also conducted (Figure 2C). The parameters of voltage dependence curves are summarized in Supplementary Table 2. The three partial LoF variants displayed apparent depolarizing shifts in both V_50_ of channel activation and channel inactivation in homomeric models; but these changes were ameliorated when co-expressed with the wild-type subunit (Supplementary Table 2). Compared with the wild-type, complete LoF variants p.A192Pfs^*^1, p.S314P, and p.P408S in heteromeric configurations exhibited similar channel activation and inactivation kinetics. Conversely, the other two variants, p.T377I and p.G379V with complete LoF showed depolarizing shifts in the V50 of channel activation, and the latter also caused a hyperpolarizing shift in the V50 of voltage dependence of channel inactivation, indicating that the two variants have dominant-negative effects on the channel dynamics of the wild-type subunit.

### Total Protein Expression and Cell-Surface Trafficking

**Figure 3:**
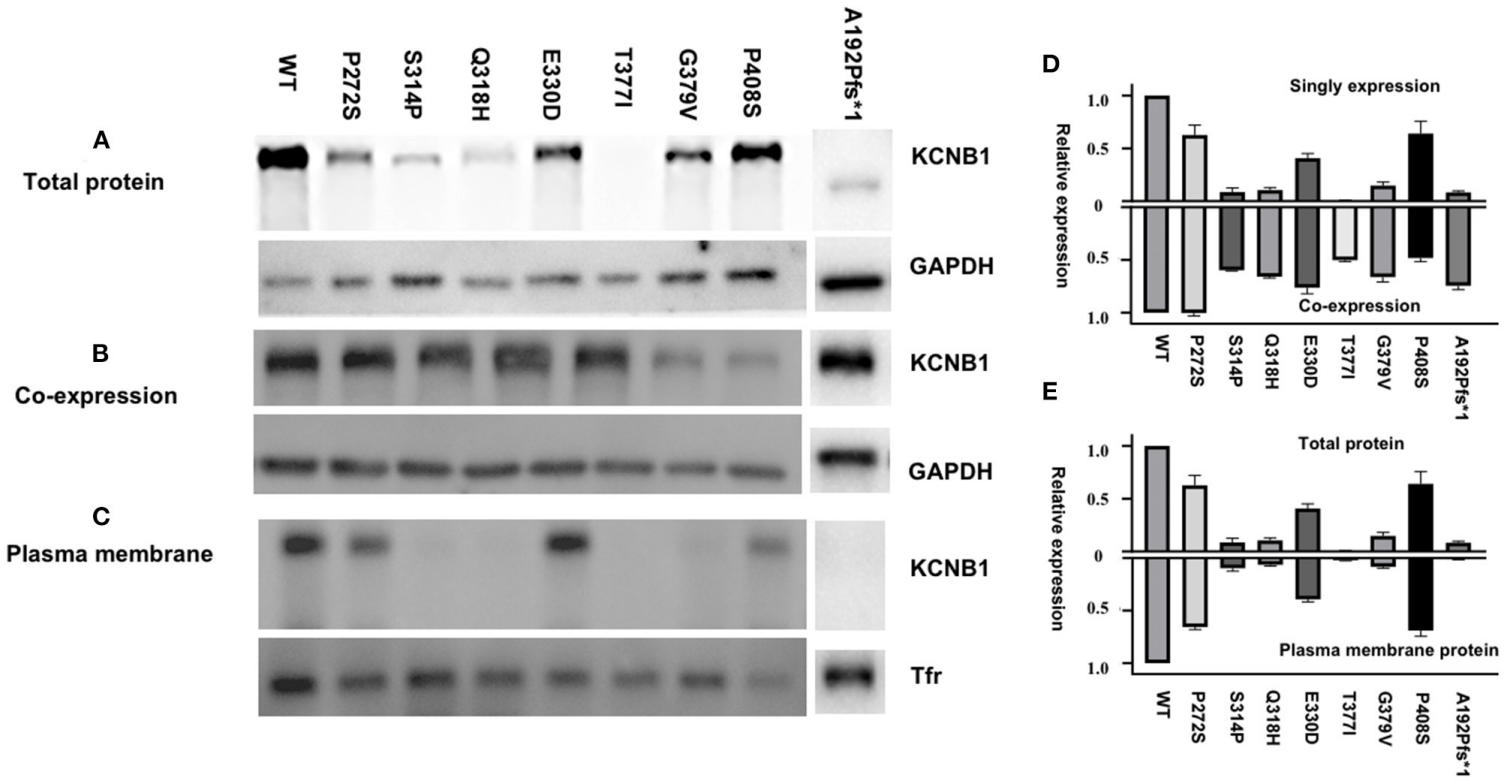
Total and cell-surface protein expression levels of *KCNB1* variants. **(A,C)** Mutant Kv2.1 proteins were expressed and trafficked to the cell surface. **(B)** Representative western blot of total protein expression of *KCNB1* variants in heteromeric models. **(D)** Comparison of KCNB1 protein expression levels of detected variants and wild-type in both configurations. **(E)** Plasma membrane trafficking efficiency of Kv2.1 channels in CHO-K1 transfected with *KCNB1* variants compared to the wild-type.

The KCNB1 protein expression of all variants was lower than the wild-type subunit in homomeric models. The complete LoF variants, expect for variant p.P408S, in the study exhibited less protein expression than partial LoF variants in homomeric configurations (Figures 3A,D). Unlike other variants, p.P408S had higher protein expression in the homologous model than in the heterologous model, indicating a dominant-negative effect on wild-type protein expression (Figures 3B,D). Noticeably, variant p.A192Pfs^*^1 led to a truncated protein due to premature stop codon but showed nearly regular KCNB1 protein expression in heteromeric channels. Variant p.A192Pfs^*^1, p.S314P, p.Q318H, p.T377I, and p.G379V displayed decreased membrane expression than wild-type, suggesting defective efficient cell-surface trafficking of these variants; while the remaining had similar surface trafficking efficiencies (Figures 3C,E).

### Comparisons of Clinical Characteristics Among Patients With Variant Effect Subgroups

Thirty-one *KCNB1* variants (23 from the published literature and eight newly reported from this study) with functional results were found (Supplementary Table 3) (7–13, 20–28). After excluding controversial variants and individuals with insufficient clinical descriptions, 33 patients with 19 complete LoF variants (Complete LoF group) and 13 patients with 6 partial LoF variants (partial LoF group), 30 patients with 16 DN effect *KCNB1* variants (DN effect group) and 18 patients with 10 non-DN effect *KCNB1* variants (non-DN effect group) were included for further analyses (Supplementary Table 3). In all, patients in complete LoF subgroup had earlier seizure onset age (64.3 vs. 16.7%, *p* = 0.007), worse seizure outcomes (18.8 vs. 66.7%, *p* = 0.025), and more kinds of seizure types (58.7 vs. 25.0%, *p* = 0.052) than those in partial LoF subgroup. Furthermore, patients older than 2-year-old in partial LoF group were more likely to have the ability to walk (90.9 vs. 60.0%, *p* = 0.077) (Table 2). Compared to non-DN effect group, more individuals in DN effect group had epilepsy (90.0 vs. 77.8%, *p* = 0.227), more than 3 kinds of seizure types (65.5 vs. 30.8%, *p*=0.039), and less achieved seizure freedom (17.6 vs. 30.0%, *p* = 0.387). Similarly, patients older than 2-year-old in the non-DN effect group were prone to walk (81.8 vs. 64.7%, *p* = 0.296) (Table 3). Patients from different subgroups exhibited indistinguishable language development levels and incidence rates of behavioral or psychiatric characteristics.

**Table 2:** Comparisons of patients with partial and complete LoF *KCNB1* variants in clinical features.

**Clinical characteristics**	**Patients with complete LoF variants**	**Patients with partial LoF variants**	***p*-value**
Patients with epilepsy	26/33 (78.8%)	13/13 (100%)	0.080
Seizure onset in the first year	18/28 (64.3%)	2/12 (16.7%)	**0.007**
≥3 kinds of seizure types	17/29 (58.7%)	3/12 (25.0%)	0.052
Seizure-free	3/16 (18.8%)	6/9 (66.7%)	**0.025**
Walk (Individual ≥ 2 y old)	12/20 (60.0%)	10/11 (90.9%)	0.077
Language development (Individual ≥ 3 y old)	8/19(42.1%)	4/10(40.0%)	0.615
Behavior problems	17/22 (77.3%)	10/12 (83.3%)	0.521

*CSWS, continuous spikes and waves during slow-wave sleep; EEG, electroencephalography; ESES, electrical status epilepticus during slow-wave sleep; ID, intellectual disability; LoF, loss of function. Statistically significant findings are bolded*.

**Table 3:** Comparisons of patients with DN and non-DN effect *KCNB1* variants in clinical features.

**Clinical characteristics**	**Patients with DN effect variants**	**Patients with non-DN effect variants**	***p*-value**
Patients with epilepsy	27/30 (90.0%)	14/18 (77.8%)	0.227
Seizure onset in the first year	15/29 (51.7%)	8/13 (61.5%)	0.401
≥3 kinds of seizure types	19/29 (65.5%)	4/13 (30.8%)	**0.039**
Seizure-free	3/17 (17.6%)	3/10 (30.0%)	0.387
Walk (Individual ≥ 2 y old)	11/17 (64.7%)	9/11 (81.8%)	0.296
Language development (Individual ≥ 3 y old)	8/15(53.3%)	6/9(66.7%)	0.418
Behavior problems	19/23 (82.6%)	8/10 (80%)	0.605

*CSWS, continuous spikes and waves during slow-wave sleep; DN, dominant-negative; non-DN, non-dominant effect; EEG, electroencephalography; ESES, electrical status epilepticus during slow-wave sleep; ID, intellectual disability. Statistically significant findings are bolded*.

## Discussion

### Clinical Characteristics in Our Cohort Are Similar to Previous Studies

To the best of our knowledge, this is the first Asian cohort study of *KCNB1*-related neurodevelopmental disorder. We reached similar conclusions from the previous reports; its clinical manifestations include variable epilepsy, global developmental delay, and behavior issues (8–10), indicating no ethnic differences. Ninety percent of patients in the study had epilepsy, 55.6% were recorded to have more than three seizure types, and 44.4% achieved seizure free, as per the last follow-up. The seizure outcomes in disorder were quite variable. It was worth noting that 5/9 cases had a history of fever-induced or fever-related epileptic events, incredibly patient 2 who was diagnosed with febrile convulsion rather than epilepsy. Unlike sodium channel genes such as *SCN1A* (29), little attention has been paid to the relationship between hyperthermia and seizure occurrence in the disease. Our findings suggest that seizures associated with fever in the early stages of the disease might be an alarm for this condition.

Based on our clinical observations and previous literature, the global developmental delay seems to be more prominent and shared in the disease, which often occurred in the first year of life (8–10). Marini et al. (21) put forward that affected patients would still have moderate or severe cognitive impairment whether the seizure is controlled or not in the long-term follow-up. Strikingly, we identified a patient with a truncated variant who had a similar developmental level as her peers (further discussion on this case can be found in the next part). About 80% of the patients in previous series had behavioral problems including autistic traits, aggression, and hyperactivity (8–10), which is consistent with our results. The typical EEG pattern in the research was slow background activity with multifocal, diffuse, or generalized epileptiform discharges. Malignant EEG patterns, including hypsarrhythmia and CSWS/ESES, were also detected in previously reported patients (8).

### Manual Patch Clamp Recording vs. Automated Patch Clamp Recording

Functional analyses of 8 novel *KCNB1* variants by manual electrophysiology were conducted in the study, while19 *KCNB1* variants using automated patch clamp recording in Kang's study (11). We compared their results with ours and found some controversies. Firstly, in our study, partial LoF variant p.E330D was classified into complete LoF variant in Kang's study, so did p.R306C and p.V378A (11, 13, 26). However, the recorded Kv2.1 currents by the traditional way is much larger than that of the new method. After careful comparison, we found that the current expression of these three partial LoF variants is <25% of the Kv2.1 wild-type. Thus this tiny current might be neglected in automated electrophysiology.

Secondly, Yu et al. proposed that the variant p.R312H has a DN effect on the voltage gate of activation, contrary to Kang's findings (11, 22). Despite the classification divergence, the variant p.E330D in our study and Kang's study had a similar assumption: DN effect (11). Based on the above analyses, these results are beyond our ability to explain why such differences exist in traditional and new methods. Further research is needed to unravel this mystery.

### The Correlations Between Variant Effects and Disease Severity

Researchers and clinicians are inquisitive about the possible link between the phenotype and genotype in single-gene inherited diseases. Therefore, introducing the molecular phenotype of genetic variants into analysis might be helpful to understand the relationship better. So far, some correlations have been unveiled. For example, the mixed dysfunctional of LoF/GoF *KCNA2* variants are associated to more severe phenotypes than simple LoF or GoF variants (30). According to our and previous researches, mutated Kv2.1 channels often result in loss-of-function of the protein. Calhoun et al. (12) reported the first partial LoF KCNB1 variant p.I199F and suggested that the degree of KCNB1 protein dysfunction is associated with disease severity. However, this view has not been well proved. Here, we concluded and compared clinical characteristics of patients with complete and partial LoF variants, DN and non-DN effect variants, and got some hallmarks about clinical manifestations among different variant effect subgroups.

Firstly, compared with that in complete LoF subgroup, patient in the partial LoF subgroup seemed to have a less severe epilepsy phenotype, with later seizure onset age, fewer seizure types, and better seizure outcomes. Moreover, patients in the DN effect group had a higher rate of epilepsy and multiple seizure types and a lower probability of obtaining seizure-free. From this perspective, our finding was in line with previous assumption that the epilepsy phenotype might be linked to the extent of Kv2.1 channel impairments caused by variants (8). However, we have not found the correlations between intellectual development or behavior characteristics and variant effects.

Even though we got some links between phenotype and genotype by analyzing the molecular phenotype of *KCNB1* variants in disorder, larger sample cohort studies and more functional research are invited to confirm these relationships.

### If It Is Not DN Effect, What Else Might It Be?

DN effect is a classical molecular mechanism in genetic diseases, which is defined as the variant that will adversely affect the co-expressed wild-type activity. Many *KCNB1* variants have disease effects in this way, such as the well-validated variant p.G379R, which interferes with wild-type functions in both potassium conductance and non-conductance aspects (7, 15). We found that not all variants could be considered to have DN effects according to their functional results. On the one hand, previous work has been mainly focused on unveiling their deficits in potassium conductance rather than their non-conductance roles (15). Here, we added an experiment on the KCNB1 protein expression in the heteromeric models, which aided us in identifying p.V408S as a DN effect variant. Thus, some *KCNB1* variants with DN effects may be masked due to lacking of sufficient experimental evidence.

On the other hand, haploinsufficiency could be another possible mechanism in the disorder. In our cohort, patient 1 with a truncated variant p.A192Pfs^*^1 almost got remission from the diseases—no epileptic seizure and normal school performances, which is the best prognosis so far. As almost all reported truncated KCNB1 variants, including ours, located in the last exon of the gene, they were presumed to generate truncated proteins rather than undergoing nonsense-mediated mRNA decay. Functional data of variant p.A192Pfs^*^1 in the study supported the assumption. Truncated KCNB1 variants have been reported to relate to the milder phenotype than missense variants, especially in Bar et al. and colleagues' study, they disclosed that there are statistical differences in epilepsy between patients with missense variants and truncated variants (8–10). The possible underlying pathophysiological explanation for such discrepancy could be that the two mutant types do not share the same molecular mechanism. Researchers uncovered that KCNQ2 variants leading to haploinsufficiency often relate to a milder phenotype—benign familial neonatal seizures, while variants with DN effect are inclined to a severer phenotype—epileptic encephalopathy (31). A similar phenomenon might also exist in patients with KCNB1 variants. Meanwhile, the current functional results of all truncated variants (p.A192fs^*^1, Y274fs^*^36, and Y533X) tended to support the mechanism of haploinsufficiency rather than the negative-dominance phenomenon (11).

### Limitations

Although we successfully applied functional data to establish phenotype and genotype correlations in the present study, there are some limitations. The biggest problem is that the functional data we used here is partly from literature, and some of them vary among different laboratories. However, those controversial data were excluded before the analyses. Secondly, all experiments in the study were performed in the CHO-K1 cell line, which could not well reflect functional alterations caused by *KCNB1* variants *in vivo*. Future studies should be taken on patient-derived induced pluripotent stem cells (iPSC) or *in vivo* to better understand the disorder's pathogenesis (32, 33).

## Conclusions

In the study, we found that patients with *KCNB1* variants in the Asian cohort have similar clinical manifestations as that of other regions: variable seizures, psychomotor developmental delay, and behavior problems. Molecular phenotypes of *KCNB1* variants through some functional analyses aid in establishing genotype and phenotype linkage in the diseases. Truncated *KCNB1* variants leading to haploinsufficiency are related to milder phenotypes. *KCNB1* variants with complete LoF and DN effect have more severe epilepsy.

## Data Availability Statement

The raw data supporting the conclusions of this article will be made available by the authors, without undue reservation.

## Ethics Statement

The study was conducted according to the guidelines of the Declaration of Helsinki, and approved by the Ethics Committee of Xiangya Hospital, Central South University, Changsha, Hunan (201605585). Written informed consent to participate in this study was provided by the participants' legal guardian/next of kin. Written informed consent was obtained from the individual(s), and minor(s)' legal guardian/next of kin, for the publication of any potentially identifiable images or data included in this article.

## Author Contributions

FY and JX: conception of the study. JX, JP, and ZL: experimental design and study designation. SC, HD, XD, LY, and BC: patient's evaluation. JX, SC, and BC: experiments. JX, HD, SC, and ZL: data acquisition and analysis. JX, MK, and FY: first version of the manuscript. JX, MK, LY, and FY: revised version of the manuscript. All authors read and approved the final version of the manuscript.

## Funding

This research was funded by the National Natural Science Foundation of China [NO. 81771408], the Graduate Innovation Program of Central South University [NO. 2019zzts338] and Hunan Province Key Field Research and Development Plan [NO. 2019SK2081].

## Conflict of Interest

The authors declare that the research was conducted in the absence of any commercial or financial relationships that could be construed as a potential conflict of interest.

## Publisher's Note

All claims expressed in this article are solely those of the authors and do not necessarily represent those of their affiliated organizations, or those of the publisher, the editors and the reviewers. Any product that may be evaluated in this article, or claim that may be made by its manufacturer, is not guaranteed or endorsed by the publisher.
