# Supplementary material for: Correlation Analyses of Clinical Manifestations and Variant Effects in KCNB1-Related Neurodevelopmental Disorder
Source: Front Pediatr. 2022 Jan 5;9:755344. doi: 10.3389/fped.2021.755344 (PMC8767024; doi:10.3389/fped.2021.755344)
Supplement: Supplementary file 2 [file Data_Sheet_1.PDF]

Table S1 Volume and number of Cell for analyzing Ipeak current density

Configurations or variants	Cell Volume Mean size ± S.E.M.(pF)	n
**WT**	12.9±2.6	41
**A192Pfs*1**	14.0±3.0	37
**WT: A192Pfs*1**	15.3±2.2	36
**P272S**	12.5±2.3	30
**WT:P272S**	13.3±2.9	33
**S314P**	12.3±3.5	28
**WT:S314P**	13.4±2.3	25
**Q318H**	13.3±2.7	37
**WT:Q318H**	13.4±2.6	30
**E330D**	13.8±2.2	40
**WT:E330D**	13.0±2.3	43
**T377I**	12.3±3.6	22
**WT:T377I**	13.6±2.8	29
**G379V**	13.9±3.7	37
**WT:G379V**	14.0±3.0	21
**P408S**	12.9±3.8	26
**WT:P408S**	12.7±2.9	24

Abbreviations: n: number

Table S2 Biophysical properties of KV2.1 variants singly or co-expressed with WT.

**Configurations or variants**	**Voltage-dependence of activation**		**Voltage-dependence of deactivation**	
	**V50**	***k***	**V50**	***k***
**WT**	6.4±2.3	14.8±4.2	-9.0±3.7	-20.1±4.8
	(n=8)	(n=8)	(n=13)	(n=13)
**WT: A192Pfs*1**	10.1±4.7	16.5±4.1	-6.4±3.9	-21.6±3.6
	p=0.49350.5075(n=9)	*p*=0.7761(n=9)	*p*=0.6348(n=8)	*p*=0.8053(n=8)
**P272S**	111.9±4.1	23.6±3.7	37.9±5.3	15.8±4.0
	p<0.0001(n=7)	*p*=0.1400(n=7)	*p*<0.0001(n=6)	*p*<0.0001(n=6)
**WT:P272S**	36.9±3.8	23.6±1.0	4.0±2.5	-23.4±3.0
	p<0.0001(n=8)	*p*=0.0768(n=8)	*p*=0.0093(n=7)	*p*=0.5672(n=7)
**WT:S314P**	2.5±2.5	12.7±3.7	-6.2±4.8	-15.2±3.9
	p=0.2703(n=8)	*p*=0.7132(n=8)	*p*=0.6517(n=7)	*p*=0.4387(n=7)
**Q318H**	57.7±4.7	23.0±3.8	8.7±3.3	12.0±3.7
	p<0.0001(n=8)	*p*=0.1699(n=8)	*p*=0.0033(n=5)	*p*<0.0001(n=5)
**WT:Q318H**	11.7±4.5	17.5±2.3	-9.7±4.7	-15.1±4.7
	p=0.3153(n=9)	*p*=0.5842(n=9)	*p*=0.9086(n=7)	*p*=0.4674(n=7)
**E330D**	78.2±3.7	26.2±1.8	7.8±2.9	10.8±2.6
	p<0.0001(n=10)	*p*=0.0327(n=10)	*p*=0.0029(n=5)	*p*<0.0001(n=5)
**WT:E330D**	49.7±4.1	22.2±2.6	-9.2±3.1	-13.8±3.1
	p<0.0001(n=7)	*p*=0.1612(n=7)	*p*=0.9674(n=7)	*p*=0.2848(n=7)
**WT:T377I**	21.0±2.6	17.6±1.5	-6.9±4.6	-13.5±3.8
	p=0.0009(n=8)	*p*=0.5461(n=8)	*p*=0.7269(n=8)	*p*=0.2945(n=8)
**WT:G379V**	17.6±3.0	18.9±1.9	-20.7±0.8	-7.7±2.5
	p=0.0122(n=7)	*p*=0.3953(n=7)	*p*=0.0086(n=5)	*p*=0.0360(n=5)
**WT:P408S**	9.6±2.9	14.4±0.8	-12.5±3.1	-17.3±4.2
	p=0.4013(n=9)	*p*=0.9279(n=9)	*p*=0.4773(n=8)	*p*=0.6657(n=8)

The word in red means *p* value <0.05.
